# Influence of external contextual factors on the implementation of health and social care interventions into practice within or across countries—a protocol for a ‘best fit’ framework synthesis

**DOI:** 10.1186/s13643-019-1180-8

**Published:** 2019-11-04

**Authors:** Alexandra Ziemann, Louise Brown, Euan Sadler, Josephine Ocloo, Annette Boaz, Jane Sandall

**Affiliations:** 1 Centre for Healthcare Innovation Research (CHIR), City, University of London, Northampton Square, London, EC1V 0HB UK; 2 King’s Improvement Science and Centre for Implementation Science, Institute of Psychiatry, Psychology & Neuroscience, King’s College London and National Institute for Health Research (NIHR) Collaboration for Leadership in Applied Health Research and Care (CLAHRC) South London, PO28, David Goldberg Centre, 16 De Crespigny Park, Denmark Hill, London, SE5 8AF UK; 3 Department of Social and Policy Sciences, University of Bath, 3 East, Claverton Down, Bath, BA2 7AY UK; 4 Department of Nursing, Midwifery and Health, School of Health Sciences, Faculty of Environmental and Life Sciences, University of Southampton, Southampton, SO17 1BJ UK; 5 Centre for Implementation Science, Health Service and Population Research Department, Institute of Psychiatry, Psychology & Neuroscience, King’s College London and NIHR CLAHRC South London, PO 28, David Goldberg Centre, 16 De Crespigny Park, Denmark Hill, London, SE5 8AF UK; 6 Kingston University and St. George’s, University of London and NIHR CLAHRC South London, 6th Floor, Hunter Wing, Cranmer Terrace, London, SW17 0RE UK; 7 Department of Women and Children’s Health, School of Life Course Science, Faculty of Life Sciences & Medicine, King’s College London and NIHR CLAHRC South London, St. Thomas’ Hospital, London, SE1 7EH UK

**Keywords:** Implementation, Innovation, Context, Spread, Diffusion, Scale-up, Healthcare, Social care, Framework, Theory, ‘Best fit’ synthesis

## Abstract

**Background:**

The widespread implementation of interventions is often hindered by a decline and variability in effectiveness across implementation sites. It is anticipated that variations in the characteristics of the external context in different sites, such as the political and funding environment, socio-cultural context, physical environment or population demographics can influence implementation outcome. However, there is only a limited understanding about which and how external contextual factors influence implementation. We aim to develop a comprehensive framework conceptualising the influence of external contextual factors on implementation, particularly when spreading health and social care interventions within or across countries.

**Methods:**

The review will use the ‘best fit’ framework synthesis approach. In the first stage of the review, we will examine existing frameworks, models, concepts and theories on external contextual factors and their influence on implementation from a variety of sectors and disciplines including health and social care, education, environmental studies and international development fields. The resulting a priori meta-framework will be tested and refined in the second review stage by analysing evidence from empirical studies focusing on the implementation of health and social care interventions within or across countries. Searches will be conducted in bibliographic databases such as MEDLINE, ERIC, HMIC and IBSS, grey literature sources and on relevant websites. We will also search reference lists, relevant journals, perform citation searches and ask experts in the field. There is no restriction to study type, setting, intervention type or implementation strategy to enable obtaining a broad and in-depth knowledge from various sources of evidence.

**Discussion:**

The review will lead to a comprehensive framework for understanding the influence of external contextual factors on implementation, particularly when spreading health and social care interventions within or across countries. The framework is anticipated to help identify factors explaining the decline and variability in effectiveness of interventions and assessing the prospects of implementation effectiveness, when spreading interventions. We do not intend to only develop another stand-alone implementation framework but one that can be used in conjunction with existing frameworks. The framework can be honed and validated in future empirical research.

**Systematic review registration:**

PROSPERO CRD42018084485

## Background

Despite many promising interventions being developed, their implementation into everyday practice is limited [1]. The process of translating research findings into widespread practice can be described in four phases: (1) basic research discoveries, (2) tests of interventions in trials, (3) implementation in pilot projects in single organisations and (4) the spread to several organisations and locations for the benefit of the whole population [2]. In the last phase, the widespread implementation across several implementation sites is often hindered by a decline in effect and variability in effectiveness across sites [3]. This leads to large parts of the population not equally or not rapidly benefitting from new or improved interventions [4].

It is anticipated that variations in the characteristics of the external context in different implementation sites can influence the implementation outcome. Such characteristics could be differences in legal, political and funding environments, health system organisation, socio-cultural contexts, the demographics of the served population, inter-organisational networks, power dynamics, historical developments, or physical environment and location. However, there is currently only a limited understanding about which and how external contextual factors influence the implementation of health and social care interventions, particularly when spreading interventions within or across countries [5].

Fewer studies have examined the influence of external contextual factors on implementation, compared to other factors such as the internal, i.e. intra-organisational context, or the content of an intervention [6]. The conceptualisation of what constitutes external contextual factors already varies considerably. This makes it difficult to establish what impact the external contextual factors would have. Some external contextual factors are specified in existing implementation science frameworks, for example, Greenhalgh et al.’s conceptual model of the Diffusion of Innovations in Service Organizations [7]; the Consolidated Framework for Implementation Research (CFIR) by Damschroder and colleagues [8]; the Exploration, Preparation, Implementation, Sustainment model (EPIS) by Aarons et al. [9]; the Context and Implementation of Complex Interventions framework (CICI) by Pfadenhauer and colleagues [10]; and Watson and colleagues’ definition of the external implementation context [11]. All these frameworks encompass different, but also overlapping, external contextual factors, and they vary considerably in their conceptualisation. Further, these studies’ underlying methodological approaches and evidence bases for developing the frameworks differ noticeably. We will build upon this growing understanding of external implementation context and aim at systematically deriving a comprehensive framework of how external context is influencing the implementation of health and social care interventions, especially when spreading interventions within and across countries.

## Methods/design

The systematic review protocol is registered in the PROSPERO international prospective register of systematic reviews (CRD42018084485). It was written according to the Preferred Reporting Items for Systematic Reviews and Meta-Analyses Protocols (PRISMA-P) guideline recommended for systematic review protocols [12]. The PRISMA-P checklist is included in Additional file 1.

### Review design

**Fig. 1:**
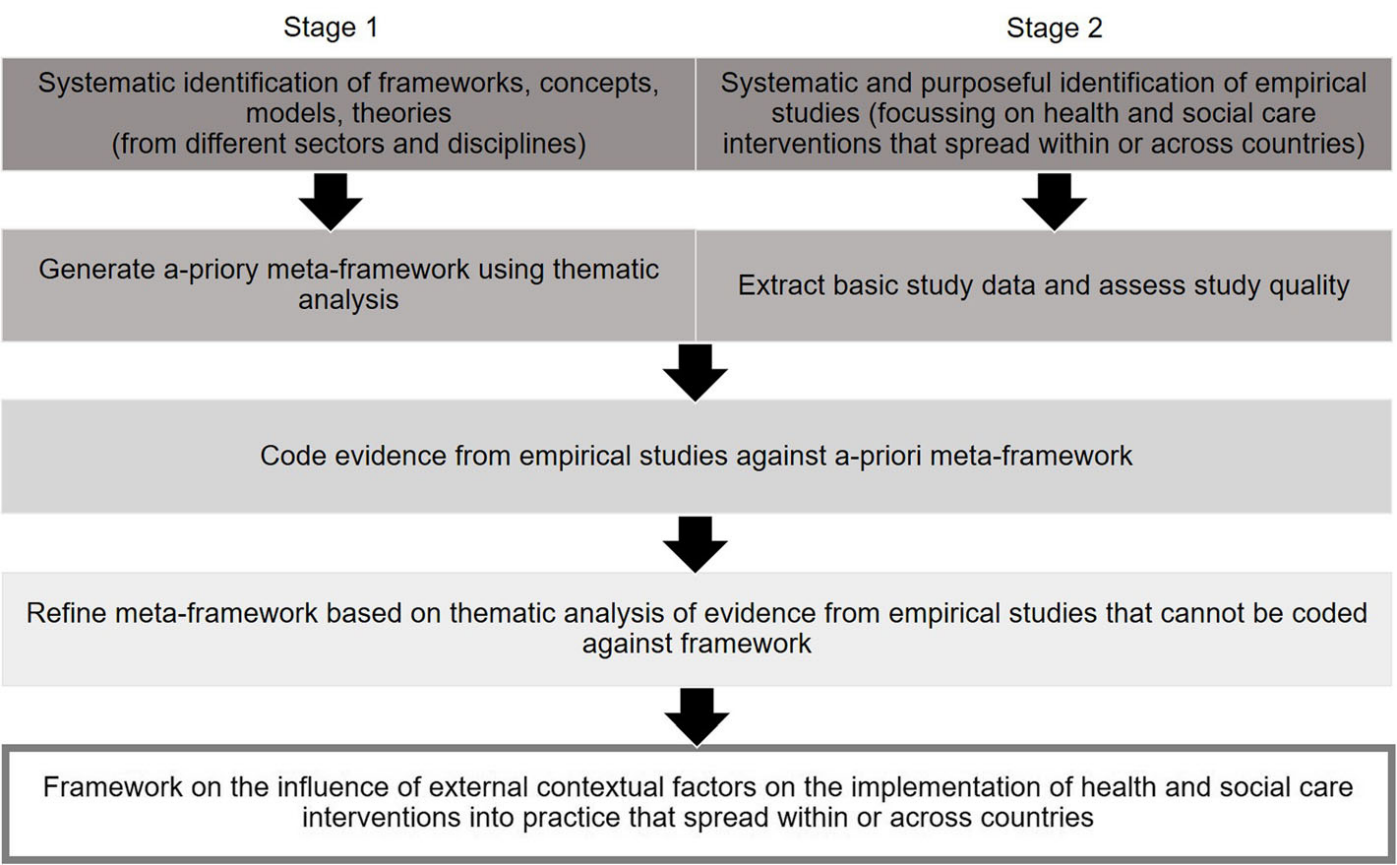
Two-stage review design following the ‘best fit’ framework synthesis approach, based on Carroll et al. [13]. Legend: The review applies a two-stage process. In the first stage, we will review existing frameworks, models, concepts and theories (stage 1—framework review). Concepts for external implementation context will be synthesised in an a priori meta-framework. In the second stage, we will test and refine the a priori meta-framework by analysing evidence from empirical studies that focus on the implementation of health and social care interventions into practice within or across countries (stage 2—empirical study review). The concepts derived from both reviews will be synthesised into a final framework

The review will follow the ‘best fit’ framework synthesis approach developed by Carroll et al. which is especially suited to develop a comprehensive framework based on existing evidence [13] (Fig. 1). The ‘best fit’ approach allows for either identifying an appropriate (or ‘best fit’) framework from the published literature to guide the thematic synthesis of evidence from empirical studies or for generating a new meta-framework by systematically searching for and synthesising published frameworks. We chose the latter approach as we did not deem any published framework to be comprehensive in terms of focusing on external implementation context.

The review is divided into two stages. Stage 1 (framework review) will follow the BeHEMoTh (Behaviour of interest, health context, exclusions, models or theories) approach to systematically identify theories, models, frameworks and concepts of external implementation context in the scientific and grey literature from different sectors and disciplines [14]. Frameworks, theories, models and concepts identified in stage 1 will be synthesised in an a priori meta-framework using thematic analysis [15]. Stage 2 (empirical study review) will apply a systematic search and purposeful sampling approach to identify information-rich empirical studies of health and social care interventions implemented into practice within or across countries. Evidence from stage 2 will be coded against the a priori meta-framework generated in stage 1. Emerging themes from empirical studies that cannot be coded against the a priori meta-framework will be incorporated into the meta-framework. The result of the review will be a refined framework on the influence of external contextual factors on implementation. This new framework can subsequently be honed and validated in future empirical research.

### Aims of the study

The primary aim of the review is to understand which and how external contextual factors influence the implementation of health and social care interventions into practice within or across countries. Each of the two consecutive review stages has specific review questions:

- Stage 1 (framework review):
  How are external contextual factors that influence the implementation of interventions into practice conceptualised within existing theory?
- Stage 2 (empirical study review):
  1. How are external contextual factors influencing the implementation of health and social care interventions into practice within or across countries?
  2. What is the evidence on this influence regarding:
    - The characteristics of the implementation process at different levels (i.e. micro, meso, macro levels), the involved stakeholders, the internal context of an organisation and the intervention?
    - Implementation outcomes?
    - Different types of interventions and different types of implementation strategies?
    - The implementation of interventions spreading internationally from one country to another compared to interventions spreading within the same country?

### Eligibility criteria

#### Types of studies

In the framework review (stage 1), we will include studies that focus on exploring, testing or developing frameworks, theories, concepts or models of the implementation of interventions. Studies describing statistical or technical, care or disease models will be excluded. The review will not be restricted to study type and will include, for example, experimental and non-experimental studies, quantitative, qualitative and mixed methods studies, hybrid implementation studies, process evaluations and conceptual studies.

In the empirical study review (stage 2), we will include primary studies analysing qualitative empirical data of the implementation of interventions. We will exclude non-empirical studies and studies not analysing the implementation of interventions, e.g. studies only analysing the effectiveness of interventions. The review will be restricted to studies presenting qualitative evidence from, for example, qualitative and mixed methods studies, hybrid implementation studies and process evaluations.

#### Domain

In the framework review (stage 1), we will include studies set in any non-profit public or private service domain such as health care, public health, social care, education, environment, public administration and international development fields. We will not only focus on studies set in the health and social care domain in this review stage but seek to include evidence from other domains such as education or international development that might be transferrable to the health and social care domain.

In the empirical study review (stage 2), studies focusing on interventions implemented in a non-profit public or private health and social care domain will be included. Studies set in for-profit businesses in the private domain will be excluded in both review stages.

#### Participants

We will include studies in both review stages that focus on participants in a practice setting, including (a) service users, i.e. members of the public who might be using the intervention, patients, carers and people from organisations that represent service users, and/or (b) service providers, including professionals and managers. Studies focusing only on participants in a policy or research setting will be excluded.

#### Intervention

In both review stages, we will include studies focusing on active intervention implementation. We will exclude studies focusing only on the stages of passive diffusion and dissemination of interventions [7]. Further, studies focusing purely on utilisation or transfer of knowledge instead of the implementation of tangible practices or interventions will be excluded.

In the empirical study review (stage 2), we will include studies describing the active implementation of a health or social care intervention within or across countries. We will exclude pilot implementation studies and planned but not yet implemented interventions. We will include studies only focusing on interventions targeting delivery arrangements of healthcare defined according to the Effective Practice and Organisation of Care (EPOC) taxonomy [16]. As we only focus on practice settings (i.e. in primary, secondary, voluntary, community care settings), studies only describing financial and governance arrangements will be excluded from our review. Social care interventions are defined as the provision of social work, personal care, protection or social support services to children or adults (or their carers) in need or at risk, or adults with needs arising from disability, illness, old age or poverty. They include working with individuals, small groups or communities and cover services provided by public bodies, the voluntary sector or accessed on a self-funded basis by the public. We will only include health and social care interventions that improve service user or provider outcomes, or the quality of services. We will exclude studies set in a non-health or non-social care domain, defined as interventions that are implemented in another domain, e.g. education, and that are not delivered by health or social care professionals. We will exclude studies focusing on the implementation of an intervention only in one single site without it having been spread to other implementation sites. We define a site by geographic location in order to capture the influence of different external contexts.

In both reviews, there will be no restriction regarding the type of intervention, type of implementation strategy [17], or level of implementation (i.e. micro, meso, macro level).

#### Context

In both review stages, we will include studies focusing on one or more external contextual factors which can be defined as characteristics of the setting surrounding an organisation in which the implementation takes place [7, 11]. Such external contextual factors could be, for example, legal, political and funding environments, health system organisation, socio-cultural contexts, the demographics of the served population, inter-organisational networks, power dynamics, historical developments, or physical environment and location. Studies focusing only on characteristics of the implementation process itself, the internal (intra-organisational) context, the stakeholders involved in the implementation process or the implemented intervention will be excluded.

#### Outcomes

In the empirical study review (stage 2), we will include studies describing the influence of external contextual factors on implementation outcome, ideally as defined by Proctor et al. [18] (Table 1). We will also include studies if they refer to other implementation outcomes, such as the utilisation of an intervention.

**Table 1:** Implementation outcome measures included in the review

Implementation outcome	Definition according to Proctor et al. [18]
Acceptability	Perception among implementation stakeholders that a given intervention is agreeable, palatable or satisfactory.
Adoption	Intention, initial decision or action to attempt to employ an intervention.
Appropriateness	Perceived fit, relevance or compatibility of the intervention for a given practice setting, provider or consumer; and/or perceived fit of the intervention to address a particular issue or problem.
Costs	Cost impact of an implementation effort.
Feasibility	Extent to which an intervention can be successfully used or carried out within a given setting.
Fidelity	Degree to which an intervention is implemented as it was intended in the original protocol or by the programme developers.
Penetration	Integration of an intervention within a service setting.
Sustainability	Extent to which a newly implemented intervention is maintained or institutionalised within a service setting’s ongoing, stable operations.

### Search strategy

For the framework review (stage 1), the search is following the iterative BeHEMoTh (behaviour of interest, health context, exclusions, models or theories) strategy which was developed by Booth and Carroll for the systematic identification of frameworks, models, concepts and theories from the literature [14]. Carroll et al. proposed to follow this strategy for the first stage of a ‘best fit’ framework synthesis [13]. The BeHEMoTh strategy comprises the following steps: (1) identifying theory from existing internal reference databases, (2) systematic database searches combining behaviour of interest (implementation) and context (external context) with terms for models or theory, (3) searches for named theories to identify key citations and (4) citation searches for identified theories in combination with the behaviour of interest.

For the systematic database search (step 2 of the BeHEMoTh strategy), we will combine generic and specific free text and database thesaurus terms for implementation, e.g. implementation, adoption, knowledge transfer, with terms for external context, e.g. external context, outer setting, structural environment and terms for theories, models, concepts and frameworks. An example of the proposed search strategy for MEDLINE (via Ovid) can be found in Additional file 2. The search covering scientific and grey literature will be performed in the following databases:

- Business Source Complete (from date of inception)
- CINAHL (Cumulative Index to Nursing and Allied Health) (from date of inception)
- Embase (from 1947)
- ERIC (Education Resources Information Center) (from date of inception),
- Global Health (from 1973)
- HMIC (Health Management Information Consortium) (from 1979)
- IBSS (International Bibliography of the Social Sciences) (from 1951)
- MEDLINE (from 1946)
- ProQuest Dissertations and Theses Global (from date of inception)
- PsycINFO (from 1806)
- SCOPUS (from 2004)
- Social Policy and Practice (from date of inception)
- Web of Science (from 1900)

In the empirical study review (stage 2), the database search is combining generic and specific free text and database thesaurus terms for external contextual factors with terms for implementation, and terms for spread within or across countries, e.g. spread, scale-up, cross-country, and multi-site. The search strategy for this review stage will additionally be informed by the results of the framework review (stage 1), e.g. regarding terms for external contextual factors. The search covering scientific and grey literature will be performed in the following databases:

- CINAHL (Cumulative Index to Nursing and Allied Health) (from date of inception)
- Embase (from 1947)
- HMIC (Health Management Information Centre) (from 1979)
- IBSS (International Bibliography of the Social Sciences) (from 1951)
- MEDLINE (from 1946)
- ProQuest Dissertations and Theses Global (from date of inception)
- PsycINFO (from 1806)
- Social Policy and Practice (from date of inception)

Besides searching electronic databases, we will hand-search reference lists of included articles and perform citation searches of included articles and authors to identify further publications linked to included studies. We will also perform citation searches for the theories identified in the framework review (stage 1) in combination with terms for health and social care interventions spread within or across countries. Further, we will search Google Scholar to cross-check that we have not missed any relevant publications.

For both reviews, we will search the grey literature databases GreyLit and OpenGrey. We will also hand-search websites of relevant institutions and organisations such as the World Health Organization, King’s Fund and the Health Foundation and relevant journals in which key articles were published, such as Implementation Science. In addition, we will ask experts in the field to identify any unpublished and ongoing work. Both reviews are restricted to publications in the English language. We will not apply any restrictions towards population, place, study type and publication year. We will include any publication type except for conference abstracts and study protocols.

### Study selection, data extraction and analysis

#### Selection

In both reviews, citations will be managed using Rayyan [19] and EndNote X9. Pairs of reviewers will independently screen the title and abstract of records and full texts for inclusion (e.g. AZ (100%) + LB (30%), ES (20%), JO (10%), AB (10%), and JS (10%)). Disagreements will be resolved by group discussion and consensus in the review team. We will calculate inter-rater reliability midway and at the end of the screening process to ensure consistency between the reviewers. We aim to improve the inter-rater reliability after the first calculation by refining the inclusion criteria in the review team.

In the empirical study review (stage 2), we follow the threefold purposeful sampling approach applied by Benoot and colleagues [20]. We chose this approach as the authors had a similar literature synthesis objective in that they aimed at constructing and refining a theory. From the eligible studies identified in the systematic search, we intend to select a sample of rich cases providing in-depth information to answer research questions 2 a–d (intensity sampling). We also apply a maximum variation sampling approach and a disconfirming sampling approach to allow for refining the external context concepts in the a priori meta-framework developed in stage 1. Based on the extracted data from eligible studies (see below), we will first select information-rich studies based on the density of information provided to answer research questions 2a–d and the quality and clarity of the studies (intensity sampling). We will then select studies that vary as much as possible from each other, for example, in study design, conceptual lens, implementation level, intervention type, implementation outcome and the described concepts of external context (maximum variation sampling). In the last step, we will identify studies describing diverging concepts of external context and conceptual lenses (disconfirming sampling). Publications on the same study will be merged. Sampling of articles will be done by one reviewer (e.g. AZ) and discussed and agreed upon with another reviewer (e.g. LB). Disagreements will be resolved by group discussion and consensus within the review team.

#### Data extraction and analysis

In the framework review (stage 1), we will develop an a priori meta-framework using thematic analysis of the included frameworks, concepts, theories and models to identify commonalities and differences [13]. Themes will be supported by descriptions or definitions from the included studies if such detail is provided. Key concepts identified in stage 1 will inform the construction of the data extraction form for the empirical study review (stage 2).

In the empirical study review (stage 2), the data extraction form for coding empirical studies will include basic information on the studies and specific information related to research questions 2 a–d such as study title, first author name, publication year, study design, study country/countries, setting, study participants/stakeholders (e.g. service providers, service users), intervention, implementation strategy, level of implementation (macro, meso, micro), implementation outcomes and if the spread of the intervention was within or across countries. Furthermore, it will include information on external context concepts and the applied conceptual lens. Finally, the data extraction form includes quality assessment criteria (see below). The data extraction form will be piloted independently by two reviewers (e.g. AZ, LB) on a sample of the studies and jointly agreed upon by all review team members. Once all appropriate data has been mapped deductively to the meta-framework a separate inductive process of thematic analysis will be used to accommodate any remaining data against new concepts within an augmented framework. One reviewer (e.g. AZ) will extract data and perform the thematic analyses, with a second reviewer (e.g. LB) validating the results by independently extracting and analysing data from a sample of the studies. Results will be discussed with all members of the review team. Disagreements will be resolved by group discussion and consensus within the review team.

### Quality assessment

We will assess the internal validity of individual empirical studies, focusing on how the design and conduct of each study has been reported following the quality appraisal approach suggested for the ‘best fit’ synthesis approach by Carroll et al. [13, 21]. We will classify studies according to the number of quality criteria they meet. If a study meets two or more quality criteria, it will be rated as being of adequate quality. If only one or no quality criterion is met the study will be rated as being of inadequate quality. We will perform a qualitative sensitivity analysis following the synthesis stage (see below) to assess how each individual study contributes to the final synthesis and how studies that were rated inadequate in terms of quality are contributing to the synthesis and how exclusion of inadequate studies would affect the synthesis.

The conceptual framework derived from the synthesis (see below) will be assessed for risk of bias in terms of selection and reporting of the evidence used to generate the framework. We will explore, for example, any unexplained absence of themes (e.g. differences between the a priori meta-framework and the final framework), the absence of negative or disconfirming evidence and the sensitivity to variables such as design, setting, participants, or frequency of reported themes in included studies [13]. The analysis of the differences between the two frameworks is also a test for a form of publication bias of the included empirical studies in stage 2, if themes are not reported in the empirical studies that were included in the a priori meta-framework.

### Data synthesis

Based on the concepts and themes identified from the two linked review stages, we will derive a new final framework [13]. In a first step, the themes identified from conceptual frameworks in stage 1 and from the empirical data in stage 2 will be incorporated within a new framework. In a second step, the evidence will be revisited to include relationships between framework themes. This process will result in a conceptual diagram and a narrative supporting the diagram that refers to the included studies.

### Amendments to the protocol

Any amendments to the protocol will be documented. Records in the PROSPERO database will be updated when important changes are introduced. All amendments to the protocol will be described and explained in the publication of the review results.

## Discussion

The review will lead to a comprehensive framework on the influence of external contextual factors on the implementation of interventions in health and social care practice, especially with a focus on interventions that spread within or across countries. The framework is anticipated to help identify reasons and factors explaining the decline and variability in effectiveness of an intervention and also assess the prospects of implementation effectiveness when spreading interventions. By improving the spread of interventions, a larger proportion of the population can more quickly and more equally benefit from new or improved services. The framework can be validated and honed through future empirical research.

We are aware of the vast number of existing frameworks in the field of implementation science [22]. This will be the first framework providing a consolidated conceptualisation of external implementation context and it can be applied when the focus of a study or implementation project is to understand external implementation context. However, we do not only intend to develop another stand-alone framework but a framework that can be used in conjunction with existing implementation theories, models and frameworks. The new framework can contribute a deeper, broader and consolidated conceptualisation of the factor ‘external context’ that is included in other existing frameworks. Another critique of the large number of existing frameworks is the lack of applicability or actual application [23]. By following a thorough, systematic approach deriving evidence from not only the theoretical but empirical literature, we are aiming at developing a framework that is applicable in practice as it is based in evidence derived from implementation practice. Further, many determinant frameworks such as the one proposed here are criticised for simply listing determinants but not reflecting on the connections between determinants or the mechanisms that link determinants with implementation outcomes [23]. Through review stage 2, we intend to derive the necessary level of detail from empirical studies to enrich the framework and make connections and causal links visible.

We chose the ‘best fit’ approach as it has shown to be suitable for the structured and transparent development of a framework based on synthesising existing evidence. The approach and especially the development of meta-frameworks have been suggested as a useful evidence synthesis approach for the field of quality improvement and implementation [15]. With its two-stage approach, it allows us to not only compile evidence from existing theory into a meta-framework but enhance the framework’s comprehensiveness and representativeness with additional evidence from empirical studies.

We are aiming to develop a comprehensive framework covering a variety of external contextual factors at multiple levels ranging, for example, from political and funding environments and inter-organisational networks to population characteristics, physical environments and historical developments. To achieve this, we decided to keep the framework review (stage 1) broad to include a wide range of existing frameworks, models, concepts and theories from different sectors and disciplines such as education, management, environmental studies and international development. Studies from these areas might contain useful concepts of external context applicable to the implementation of health and social care interventions. Further, we will follow a broad search strategy covering a large amount of scientific and grey literature sources and covering published and unpublished work. We will not restrict the review to any type of evidence or study design. There is also no restriction regarding, for example, a specific type of intervention, setting or implementation strategy. This strategy will enable us to obtain a broad knowledge of external contextual factors and their influence on implementation processes and outcomes. Nevertheless, the quality of studies and their impact on the findings will be evaluated through the quality assessment and sensitivity analysis.

Our broad approach in the review poses the risk of a large number of potentially eligible studies and an unfeasible workload during the screening and data analysis process. We have therefore chosen to restrict the database search for the stage 1 review by focusing on studies that contain the terms for framework, model, theory or concept in the title only. We will limit the risk of missing relevant studies by applying several additional search steps, including searching for grey literature, citation search, hand-searching references and relevant journals, and asking experts in the field.

Furthermore, we have chosen to focus the stage 2 review to qualitative empirical studies describing health and social care interventions that spread within or across countries. This allows us to capture empirical studies with a higher potential to describe the impact of external contextual factors on implementation compared to studies focusing on single implementation sites. In addition, the stage 2 review is not restricted to a certain group of interventions or implementation strategies enabling us to still capture a broad range of external contextual factors and their influence on implementation. The threefold purposeful sampling approach also helps us to gather both in-depth and comprehensive information on the role of external contextual factors.

We have appointed an international external advisory board for quality assurance including academic experts in health and social care, contextual factors, implementation and the ‘best fit’ review methodology. Additionally, we have appointed professional and service user/carer representatives with the aim of including perspectives beyond those of researchers. The professional representative was appointed based on expertise in spreading or adopting health and/or social care interventions transferred from elsewhere. The service users/carers have been appointed to provide their perspective on and experience with factors affecting their use of newly implemented health and social care interventions. The advisory board was and will be consulted and asked to comment on the review methodology and (preliminary) results, the protocol, publication manuscripts and for any specific queries arising during the review process.

## Supplementary information

**Additional file 1.** Prisma-P 2015 checklist.

**Additional file 2.** Search strategy – Framework review (review stage 1) – MEDLINE (OVID).

## Data Availability

Not applicable

## Abbreviations

BeHEMoTh: Behaviour of interest, health context, exclusions, models or theories
CFIR: Consolidated Framework for Implementation Research
CICI: Context and Implementation of Complex Interventions
CINAHL: Cumulative Index to Nursing and Allied Health
EPIS: Exploration, Preparation, Implementation, Sustainment
EPOC: Effective Practice and Organisation of Care
ERIC: Education Resources Information Center
GreyLit: Grey Literature Report
HMIC: Health Management Information Consortium
IBSS: International Bibliography of the Social Sciences
PRISMA-P: Preferred Reporting Items for Systematic Reviews and Meta-Analyses Protocols
PROSPERO: International prospective register of systematic reviews
