# Supplementary material for: Influence of external contextual factors on the implementation of health and social care interventions into practice within or across countries—a protocol for a ‘best fit’ framework synthesis
Source: Syst Rev. 2019 Nov 4;8:258. doi: 10.1186/s13643-019-1180-8 (PMC6827205; doi:10.1186/s13643-019-1180-8)
Supplement: Supplementary file 2 — Additional file 2. Search strategy – Framework review (review stage 1) – MEDLINE (OVID). [file 13643_2019_1180_MOESM2_ESM.pdf]

Additional File 2

Search strategy - Framework review (review stage 1) - MEDLINE (OVID)

1. translational medical research.sh
2. evidence based practice.sh
3. diffusion of innovation.sh
4. knowledge utili*.ab,ti
5. knowledge mobili*.ab,ti
6. knowledge transfer*.ab,ti
7. knowledge translat*.ab,ti
8. implement*.ab,ti
9. adopt*.ab,ti
10. research utili*.ab,ti
11. spread*.ab,ti
12. scale-up.ab,ti
13. scaling-up.ab,ti

14. external context*.ab,ti
15. external environment*.ab,ti
16. outer context*.ab,ti
17. outer setting*.ab,ti
18. structural context*.ab,ti
19. structural environment*.ab,ti
20. wider context*.ab,ti
21. wider environment*.ab,ti
22. wider setting*.ab,ti
23. broader context*.ab,ti
24. broader environment*.ab,ti
25. macro-level.ab,ti
26. micro-level.ab,ti
27. system-level.ab,ti
28. local context*.ab,ti
29. local environment*.ab,ti
30. regional context*.ab,ti
31. regional environment*.ab,ti
32. national context*.ab,ti
33. national environment*.ab,ti

34. framework*.ab,ti
35. concept*.ab,ti
36. theor*.ab,ti
37. model*.ab,ti

38. 1 or 2 or 3 or 4 or 5 or 6 or 7 or 8 or 9 or 10 or 11 or 12 or 13

39. 14 or 15 or 16 or 17 or 18 or 19 or 20 or 21 or 22 or 23 or 24 or 25 or 26 or 27 or 28 or 29 or 30 or 31 or 32 or 33

40. 34 or 35 or 36 or 37

41. 38 and 39 and 40
